# Relapse prevention in UK Stop Smoking Services: a qualitative study of health professionals' views and beliefs

**DOI:** 10.1186/1472-6963-9-67

**Published:** 2009-04-24

**Authors:** Shade A Agboola, Tim J Coleman, Ann D McNeill

**Affiliations:** 1 UK Centre for Tobacco Control Studies, Division of Epidemiology & Public Health, University of Nottingham, Nottingham, UK; 2 UK Centre for Tobacco Control Studies, Division of Primary Care, University of Nottingham, Nottingham, UK

## Abstract

**Background:**

NHS Stop Smoking Services in the UK provide cost effective smoking cessation interventions, but approximately 75% of smokers who are abstinent at 4 weeks relapse to smoking by 12 months. This study aimed to explore health professionals' understanding of relapse prevention interventions (RPIs), the feasibility of offering such support and whether and how these are currently used in UK NHS Stop Smoking Services.

**Methods:**

Sixteen health professionals working in UK NHS Stop Smoking Services, selected from those attending a national conference were interviewed and Framework Analysis was used to identify recurrent key themes and concepts in their perceptions and experiences of providing relapse prevention interventions (RPIs).

**Results:**

Interviewees had diverse perceptions of relapse prevention as a concept. Whilst relapse prevention was largely seen as support to prevent abstinent smokers from relapsing to smoking, some interviewees stated that RPIs were being delivered to lapsed or relapsed smokers. Current provision of RPIs was most commonly described as behavioural counselling being offered predominantly after completed cessation treatment, often in the format of 'rolling groups' which the client was encouraged to attend. Commonly identified barriers to the introduction of RPIs were funding and government targets focussed on short term cessation, smokers' low uptake of offered RPIs and an uncertain evidence base for their effectiveness. Interviewees were positive about the potential use of pharmacotherapy for relapse prevention, but were negative about the possibility of introducing proactive telephone counselling for this purpose.

**Conclusion:**

There is currently no shared understanding of the concept of relapse prevention amongst this sample of health professionals working in UK NHS Stop Smoking Services. For RPIs to be systematically delivered via these services, a commonly-held, widely-accepted and understood definition of relapse prevention would be needed. Other barriers towards introducing RPIs, such as their weak evidence and the short term cessation-focussed targets against which UK stop smoking services are measured, would also need addressing and interventions which are acceptable to abstinent smokers would need to be developed.

## Background

NHS Stop Smoking Services were established from 1999 onwards across the UK to provide support for motivated smokers who wish to quit. The effectiveness of these services has been demonstrated: for example, more than half of English services' clients achieve validated abstinence from smoking for at least four weeks and around 15% do so for at least a year[1]. Services are also very cost-effective, achieving an average cost per life year saved, after allowance for future health care savings, of £438 [2], which is over 50 times below the English National Institute of Health and Clinical Excellence's benchmark cost-effectiveness figure of £30,000 [3]. Services' clients are offered pharmacotherapy and behavioural therapy in group or individual settings [4], but as the figures above suggest, quitters' rates of relapse to smoking are high, with around 75% of those abstinent at 4 weeks after their quit date re-starting smoking by one year [1].

If stop smoking services provided abstinent smokers who are making quit attempts with effective treatments to reduce their rates of relapse to smoking (relapse prevention interventions, RPIs), then long term prolonged cessation rates could, potentially, be greatly improved. However, the evidence base for using RPIs is weak; a 2006 Cochrane review of pharmacotherapy, extended treatment interventions and interventions designed to provide smokers with skills to avoid relapse to smoking, found no evidence that any of these approaches worked for relapse prevention, although the evidence base was small [5]. As part of an investigation into the feasibility and potential effectiveness of introducing RPIs into the NHS, this evidence base is being updated and attitudes to the potential use of RPIs are being investigated. We report here the findings of a qualitative study which investigated the views, knowledge and understanding concerning RPIs of health professionals working in UK NHS Stop Smoking Services and also their experiences of providing RPIs in clinical practice. Factors which could hinder or encourage the efficient provision of RPIs in UK NHS Stop Smoking Services were also investigated.

## Methods

### Design

Health professionals working in UK Stop Smoking Services attending a UK smoking cessation conference in 2007 were asked to indicate their willingness to be interviewed at a later date on relapse prevention and 23 professionals provided contact details. All 23 were emailed seven months after the conference, invited to re-confirm their willingness to take part and agree a convenient time to be interviewed. Non-respondents to the email were contacted via telephone two weeks later. A literature review identified issues of potential importance to RPIs and, using these, a semi-structured interview schedule, with prompts (Appendix 1) was developed. Throughout interviews, open-ended questions were used to encourage participants to answer freely and these were conducted by SA via telephone in January and February 2008. Interviews covered the following subjects: knowledge and understanding of relapse prevention, types and duration of RPI provided and barriers and challenges encountered. Each interview lasted 20–25 minutes and all were audio-taped and transcribed verbatim.

### Analysis

Table 1 summarises the process of data analysis. The interviews were analysed using the Framework Method [6]. The first stage of analysis involved identifying initial themes or concepts. The themes and categories on which the analysis is based were derived from the data, rather than being imposed by the researchers [7]. The definition of emergent themes and categories were checked against the data, and subsequently refined in an iterative process [8]. Themes and sub-themes were given unique codes and a manageable index was constructed. The index was subsequently applied to the raw data, and the references were noted in the margins of the transcripts. The next stage involved constructing charts with rows and columns for each of the main themes and sub-themes that emerged. This process of "charting" allows allocation of the main themes to each column on the chart, and each interview transcript is assigned to a particular row. It ensures that enough data and context are included in the charts such that the analyst is not required to go back to the transcribed data to understand the point being made [6]. Each interview stays in the same location on every chart. After charting all the interviews, interview text relating to the research aims and objectives was collated from the themes and sub-themes. Finally, AMc and TC read a sample of ten randomly selected transcripts and confirmed that the transcripts were coded consistently and they contained data that supported the key findings of the study. Any disagreements were resolved by discussion.

**Table 1:** Process of data analysis

SA reads all transcripts in an iterative process to identify themes and sub-themes (variations in advisor experiences of providing relapse prevention treatments and variations in thinking within themes)
SA designs a framework (index) with themes and sub-themes and their working definitions

TC and AMc agree working definitions for the emerging themes and sub-themes

SA codes the transcripts for the themes. Data relating to each theme is assembled. TC and AMc independently read 10 randomly selected transcripts to see if they are being coded consistently

Working definitions for themes and sub-themes are refined. The framework (index) is re-structured to reflect the changes

SA re-codes the transcripts using the refined definitions. TC and AMc check reliability of the data and interpretation of the findings at each stage of the process

## Results

### Response

Of the 23 individuals who had indicated their willingness to participate in the study, four could not be contacted and three potential participants indicated that they no longer wished to participate, so a total of 16 interviews were conducted (12 with females). Fifteen interviewees were smoking cessation professionals who were also responsible for overseeing the day-to-day activities of their respective services or actively involved in managing one aspect of the service in which they worked and one was a regional tobacco control lead. We report our interpretation of health professionals' accounts below. Quotes from interviewees appear in Tables Three to Five and they are attributable to individual interviewees by code numbers.

### The themes

Three broad themes emerged from interview data: beliefs, knowledge and understanding of relapse prevention; RPIs for abstinent/lapsed and relapsed smokers; and, barriers and challenges. A summary of the themes and sub-themes is shown in Table 2.

**Table 2:** Index of themes and sub-themes

**Beliefs, knowledge and understanding of relapse prevention** Prevention of lapses Treatment of lapses Treatment of relapsed smokers
**Relapse prevention support for abstinent/lapsed and relapsed smokers**
***Support for abstinent/lapsed smokers***
*Content*
Behavioural counselling & pharmacotherapy
Telephone follow up
Social Activities
*Uptake*

***Support for relapsed smokers***
*Content*
Rolling groups
Recycling – fresh quit attempt
*Uptake*

**Barriers and challenges** Funding Government targets Paucity of information about effective relapse prevention interventions

### Beliefs, knowledge and understanding of relapse prevention

Interviewees had diverse perceptions of relapse prevention as a concept and shared no common definition of what this should entail (see Table 3). Their definitions of relapse prevention mostly appeared to be informed by the ways in which the cessation services for which they worked currently attempted to help abstinent smokers to remain stopped and achieve long term abstinence. Interviewees believed that relapse prevention should be mainly provided for abstinent smokers to help them remain smoke-free and **prevent lapses **by extending their acute treatment Some health professionals also believed that RPIs could be used for the **treatment of lapsed **(i.e. people who had smoked one or two cigarettes) or **relapsed smokers **(i.e. people who were now smoking regularly again).

**Table 3:** Beliefs, knowledge and understanding of relapse prevention

***Prevention of lapses*** *"Relapse prevention is making sure that, to try and stop people lapsing back to smoking, that's what I understand relapse prevention would mean"*. *"It is some action, some clinical action that the practitioner takes in order to prevent the patient from in the first place lapsing that automatically implies that it would help prevent relapse". 12* *"Relapse prevention to me is, we have an open clinic, we see them initially for at least seven weeks, but it doesn't finish at either at six or seven weeks, they can come for as long as they like". 13*
***Treatment of lapses*** *"Well it's for people who have tried to stop smoking and have lapsed, and they don't want to go back through the whole system again, they just need something to get them back on track again," 10* *"I've always felt that it's enabling clients to remain quit in the long term.......so that at the point where they've had one lapse, they actually get support to prevent them from turning into one great big relapse". 2*.

***Treatment of relapsed smokers*** *"It's really about people who have relapsed to smoking, regularly, and have gone into full-blown relapse, where they are now smoking regularly, and they need help to actually stop". 1*

#### Prevention of lapses

Most participants understood/thought that relapse prevention involved preventing lapses by providing treatment for longer than a 'usual' seven to eight week period, believing that, irrespective of the type of intervention used, offering acute cessation treatment for longer periods would ensure that more smokers remained abstinent. Almost all participants stated that relapse to smoking was most frequent when smokers ran out of medication, which perhaps explains the strong belief that extending treatment would prevent lapses. The treatment was often either group or individual behavioural support, and was extended beyond the usual timeframe for which acute cessation treatment is provided.

#### Treatment of lapses

A small number of interviewees described relapse prevention as providing support for smokers who have already had a minor 'lapse' (i.e. smoked a few cigarettes during an otherwise abstinent period) to prevent subsequent complete relapse. Interviewees believed that a single lapse could be sufficient to precipitate a later, complete relapse and that managing these would substantially reduce the risk of smokers returning to regular smoking. Interviewees, again, provided a content-centred definition for relapse prevention as being the treatment of lapses citing the provision of behavioural support and pharmacotherapy as potentially effective for those who had smoked a few cigarettes during an otherwise abstinent period.

#### Treatment of relapsed smokers

Interviewees also described relapse prevention as providing treatment for smokers who have fully relapsed to smoking. They define this, again, in terms of the content of the relapse prevention intervention and reveal that this usually involved encouraging the client to begin a fresh quit attempt and begin acute cessation treatment again or attendance at 'rolling groups' (defined below).

### Relapse prevention interventions for abstinent/lapsed and relapsed smokers

Interviewees described RPIs for abstinent/lapsed and relapsed smokers in terms of the types of support which could be provided and these differed for the two groups (see Table 4). They also reflect on the uptake of such support when offered within their services.

**Table 4:** Relapse prevention interventions for abstinent/lapsed and relapsed smokers

**Support for abstinent/lapsed smokers** **Content** ***Behavioural counselling & pharmacotherapy*** *"We run a group, so they can come in for a series of sessions which cover general healthy lifestyle, like healthy eating, getting advice, and stress reduction". 6*. *"Well everything that is involved in behavioural support, going through every situation they may encounter, preparing themselves for that, looking at tactics to cope with situations....if they've got anything in particular they're worried about, we will approach it in a practical but relaxed manner". 2* *"We work with people, whatever their issues/triggers are, we would deal with, it doesn't matter what it is, if its debt management, crisis resolution, if in quitting smoking they've got problems that need dealing with, we would deal with it". 5* *"I would strongly recommend that (pharmacotherapy) because as I said, a lot of patients reported that they actually relapsed right after they are not provided with medications". 3* "*I think it (pharmacotherapy) would be fantastic".5* ***Telephone follow up*** *"We do telephone follow-ups, say between six and twelve months, just to ask them how they are getting on, and to let them know they can access the service again and again, at any point they need". 18* *"The problem you have there is if you are going to phone everybody, you have to have the manpower, the resources to do that, from a resource point of view, I wouldn't have time to phone everybody at the moment". 13* *"It would be very hard for us to that, to phone everyone up would be ideal but impossible at the moment".15* ***Social activities*** *"We do use interventions such as diversion therapy, by getting people into community groups and community support....we don't send them home to continue sorting out themselves, we get them out in the community, get them busy and get them involved in things, they need to be busy and out there and feeling useful... we get them to go to care homes and just you know, do peoples' hair and make them cups of tea, its just engaging them with whatever involves them". 5*
**Uptake** *"I think the picture is actually, people poorly attend relapse prevention. Because they feel once they've actually reached the quit, they don't need any help...a lot of smokers don't want you chasing them up, a lot of them are fed up if you do contact them". 6* *"We used to, a couple of times a year, we'd put on like an open session, and invite everybody who'd been to the group in the last year, we'd put on a bit of food and make it a social event, and do some stuff about staying quit, but very few people attended, so it tended to be a waste of our time, so we didn't continue it". 16*

**Support for relapsed smokers** **Content** ***Rolling groups*** *"We don't have any formal relapse groups, but there are no barriers to re-entry of the service, for example in my 12 week group, drop-in group, people can come along who have relapsed and re-join the group again". 12* *"We have a rolling group that is open for anybody who wants to come back in at any time". 14* ***Recycling*** *"Yes, it's a new quit attempt isn't it? (For relapsed smokers). You would go over the reasons for relapse and then you need to go through the whole process of another quit attempt". 16*

**Uptake** *"A lot of people would rather, even though you've built up a rapport, struggle than bother you, so they think, oh no, I've failed now, they may have had one, two cigarettes, they don't contact you, so that tends to be a problem.". 8* *"I think, when you, from my experience as an ex-smoker, if you're trying to give up, and you've slipped up, and then somebody is ringing you, you think Oh God no, it's that woman again, and feel really bad". 13* *"The trouble is when people do relapse there's sort of, they're embarrassed to come back". 15*

#### Support for abstinent/lapsed smokers

##### Content

###### Behavioural counselling & pharmacotherapy

Interviewees revealed that behavioural counselling was one of the most favoured forms of treatment used to help smokers who have quit or who have had one or two brief lapses remain smoke-free. The treatment is usually provided on an individual or group basis and these smokers are helped to identify situations and triggers that might lead to smoking lapses or relapse, taught strategies to help overcome the associated urge for a cigarette, and steps to take to prevent lapses, and the case of those who have had one or two lapses, steps to prevent full blown relapse. They are also helped to understand that relapse is a spontaneous, unplanned thought process or phenomenon which is often triggered by external factors such as holidays, bereavement, and unexpected personal or financial difficulties, and are equipped with strategies to deal with these. Few individuals spontaneously mentioned the use of pharmacotherapy to prevent relapse but all were asked to comment on the feasibility of using pharmacotherapies for relapse prevention (given emerging evidence on its effectiveness, see below) and most interviewees were positive about this. (Table 4).

###### Telephone follow up

Some interviewees reported that their services provide telephone follow up calls or text messaging to support abstinent clients who had completed acute cessation treatment. Advisors kept in touch with abstinent smokers via telephone to provide motivation and support, when needed, and to help them remain smoke-free with relatively short calls (< 10 minutes) made at times specified by clients as convenient. During the call, the advisor reiterated the advantages of remaining smoke-free and reminded clients to contact the service once he/she experiences the urge to smoke a cigarette. Interviewees were quick, however, to point out that proactive telephone counselling for relapse prevention was logistically difficult and, as many clients found the calls intrusive, not a favoured intervention. They believed calls could be an unwelcome reminder for smokers that abstinence could be difficult to maintain (Table 4).

###### Social Activities

Interviewees talked about using 'diversion therapy' as a relapse prevention intervention for abstinent smokers. This was often reported to involve engaging clients in activities designed to take their minds off smoking, and provide them with feelings of well-being and importance, Activities were specially organised and could be conducted by the smoking cessation service or in community or leisure centres. Regular group activities could be lead by an abstinent smoker and those described included baking/cooking, community services and visiting hospices or nursing homes to lend support to residents.

### Uptake

Interviewees revealed that where RPIs had been offered, most clients did not take advantage of them and it was difficult to keep providing them. They admit that an important focus in the management of smoking relapse should be to help the smoker overcome such emotions. Interviewees' accounts suggest that making the client aware early on in the quit attempt of the possibility of relapse further down the quitting process may prepare the smoker for relapse and diminish the feelings of disappointment they may suffer.

#### Support for relapsed smokers

##### Content

###### Rolling groups

In some services, smokers who had already been treated but had relapsed to smoking were able to access 'rolling groups'. Health professionals described this as an 'open door' policy, with relapsed clients always free to return to the service and re-join support groups. 'Rolling groups' would not have fixed start or completion dates, so clients wishing to re-enter the service for help would be able to do so. Groups were perceived as able to provide clients the opportunity to mix with individuals who had received acute cessation treatment, relapsed, but had been able to become abstinent again. The groups are often led by a trained advisor and the sessions focus on, in addition to other issues, helping the smokers deal with circumstances that might lead to relapse.

###### Recycling – fresh quit attempt

In other services smokers who have suffered a full blown relapse were encouraged to re-enter the service as a fresh quit case. According to these health professionals' accounts, once a person has fully relapsed to smoking, relapse prevention is no longer appropriate and the individual is classified as a fresh quit attempt and begins the process of quitting smoking all over again.

### Uptake

This process of re-cycling is often hampered by the unwillingness of the smoker to admit to relapse. Health professionals' accounts suggest that providing RPIs was made difficult by service users' profound feelings of failure and embarrassment after relapsing, plus reluctance to admit to relapse. Clients generally accessed services for cessation treatments and they might not view Stop Smoking Services as an appropriate place to seek help with lapses and brief relapses.

### Barriers and challenges to using relapse prevention interventions

Funding and pressure to meet Government targets which are focussed on short term cessation and paucity of information as to the most effective relapse prevention interventions were repeatedly identified as challenges to the provision of RPIs (see Table 5).

**Table 5:** Quotes relating to barriers and challenges

***Funding*** *"We used to have a relapse prevention session, wherein we invite everyone that came to our service to attend these clinics, but we don't do that anymore because of financial pressures". 3* *"I think most PCTs would be prepared to pay for a course of treatment, but not an extended course of treatment, that's why they cut down to stop methods unlikely to be funded locally". 4*
***Government targets*** *"If you've got very busy clinics and you have Department of Health Targets to meet, you know, there's always a bit of a squeeze, in terms of how much time you've got for people to see you beyond their successful four week quit". 7* *"I think you know, the fact that we are so target driven, and the fact that reporting successes is at a month rather than if it was three months or something like that, you know the whole drive would be to see patients longer......although I think targets are probably a good thing overall, because it does focus you on hitting the three pots and all the rest of it, but I think it can be a bit counter- productive". 14*

***Paucity of Information about effectiveness of RPIs*** *"I don't think we've got anything in black and white, to be honest". 1*

#### Funding

Nearly all health professionals stated that drug budgets and funding constituted major obstacles to the introduction of RPIs for smokers. A number of interviewees revealed that they stopped providing RPIs due to a lack of funds for additional support beyond that provided during cessation treatment. Interviewees were positive about providing these interventions for motivated smokers if health authorities allocated adequate funding.

#### Government targets focussed on short term cessation

Interviewees' accounts suggested that the need to meet Government targets for NHS Stop Smoking Services exerts considerable pressure on them. Even for health professionals who were interested in and willing to provide RPIs, the pressure to achieve short term cessation for smokers (i.e. 4 week quits) often reduced the amount of time and resources that could be devoted to RPIs. Interviewees recognize this conflict and repeatedly identified this as a noteworthy barrier to the provision of RPIs within their services.

#### Paucity of information about effective relapse prevention interventions

A lack of available evidence on the effectiveness of RPIs was perceived as a barrier to their use by some participants. It was believed that it would be easier to provide these interventions for motivated smokers if there was readily available and accessible information in the literature as to the most effective RPIs. Many indicated that they would be able to integrate RPIs into their mainstream service, but only if there was sufficient evidence regarding the effectiveness of these interventions.

## Discussion

### Summary of findings

This sample of health professionals working in UK NHS Stop Smoking Services do not appear to have a shared understanding of what interventions aimed at preventing relapse to smoking should encompass. Interviewees' used their experiences to conceptualise relapse prevention and their beliefs and understanding of this were often based on the kinds of support that services in which they worked offered as relapse prevention to clients. Interviewees believed that introducing relapse prevention support into NHS Stop Smoking Services would be hindered by current performance targets which are focussed on achieving short term abstinence from smoking, following cessation treatment. There had been low uptake of RPIs in services which previously offered this and interviewees were negative about introducing additional, proactive telephone support counselling as an RPI, but were much more positive about the possibility of using pharmacotherapy for this purpose.

### Important emergent issues

Interviewees were generally positive about the potential for using RPIs for smokers accessing stop smoking services, but their varied descriptions of interventions indicated that they did not share a common definition of what these interventions should involve. Some believed relapse prevention should be aimed at lapsed smokers but, more commonly, it was perceived appropriate for preventing relapse among smokers who were still abstinent and attempting to quit. Often no distinction was made between relapse prevention treatments and routine cessation treatment. It is, perhaps, unsurprising to find differences of opinion about RPIs amongst health professionals, because the scientific literature on the subject describes studies which appear to conceptualise relapse prevention in different ways too. A Cochrane review described relapse prevention interventions as those which explicitly seek to reduce relapse rates after an acute treatment phase is successfully completed, or at some time after the date of the quit attempt [5]. However, studies in this review and which satisfied this definition include some which randomised abstainers and provided behavioural treatments [9,10] and/or drug treatments used for extended periods [11,12] and studies which randomised smokers and provided behavioural RPIs to help prevent relapse alongside standard cessation oriented therapy [13,14]. This latter approach is consistent with Marlatt and Gordon's model of relapse which predicts that relapse may occur when ex-smokers encounter high risk situations and employ inadequate coping responses to avoid smoking [15]. The behavioural counselling for relapse prevention provided would, therefore, focus on developing smokers' coping skills prior to, or early on within, a quit attempt.

Our findings suggest that health professionals would be comfortable with the notion of delivering RPIs to abstinent smokers, but the limited evidence base for the effectiveness of such interventions would need to be strengthened before this could occur. If this were to happen and RPIs were to be used in NHS Stop Smoking Services, interviewees believed that pharmacotherapy used as extended treatment for relapse prevention could be easily integrated into current routine practice, as long as costs were addressed. However, there was little or no enthusiasm for the use of proactive telephone counselling to help prevent relapse because it was considered that few abstinent smokers would welcome this type of contact and it would be logistically challenging for services to undertake.

Current performance management targets are a substantial barrier to the introduction of relapse prevention support in the UK National Health Service (NHS). For example in October 2002, the English Department of Health (DH) stated an aim that 800,000 smokers would successfully quit following help from smoking cessation services by 2006 [16] and this was subsequently translated into an aspiration that services should treat at least 5% of their local population of smokers in the course of a year, with an expected success range of 35% – 70% at four weeks [17]. For these targets, success involves smokers achieving a four week period of abstinence, so there is considerable pressure on services to achieve a high throughput of smokers who manage to stop smoking for at least one month. Interviewees' comments reflected this perceived pressure and indicated that the provision of RPIs could not be a priority for services without a change in the targets against which they are measured.

Interviewees were also concerned about the low uptake of RPIs which had often led to these being withdrawn after their introduction and was perceived as a major potential barrier to the successful introduction of RPIs into the NHS. Interviewees thought that relapsed smokers were embarrassed about their 'failure' and hence did not return for treatment. This view is consistent with the literature; lapsed smokers often feel guilty [18] and experience negative affect and decreased confidence in their ability to quit [19].

### Strengths and Limitations

This is, to our knowledge, the first qualitative study to explore smoking cessation professionals' experiences of and beliefs about providing RPIs for smokers. As interviewees all worked within the UK NHS Stop Smoking Services and as these have a strong focus on cessation treatment, findings have highlighted relevant issues that could hinder or facilitate the introduction of RPIs into this environment. Our sample was small and it is possible that by interviewing a larger, more diverse group, we would have found other important factors relating to the use of RPIs, however, similarities inherent in the accounts we obtained, suggest that we have identified most of the major issues. Also, as our sample were volunteers from those attending a smoking cessation conference, one might expect their views on relapse prevention interventions to be better informed than those of others working in smoking cessation.

## Conclusion

Should effective RPIs be identified, it is clear that health professionals would be positively orientated towards delivering these via UK NHS Stop Smoking Services. However, this process would be helped by the development of a clear definition of what relapse prevention is and should involve and it seems very unlikely that RPIs could be successfully introduced without current targets for performance management being altered to reward longer term cessation. Potential RPIs should be piloted for their acceptability to smokers making quit attempts to ensure maximal uptake of those which are introduced and it would be unwise to ignore the views of those working in stop smoking services when deciding upon the logistics of intervention delivery.

## Competing interests

Within the last five years Tim Coleman has undertaken consultancy work for Pierre Fabre Laboratories, France and also Johnson & Johnson. Both companies produce nicotine replacement therapy. The other authors declare that they have no competing interests.

## Authors' contributions

All authors designed and contributed to the write up the study and read and approved the final manuscript. SA carried out the interviews as described in Box 1 and wrote the first draft of this article. SA is the guarantor for this manuscript.

## Appendix 1

### The interview schedule

**1 **What interventions are currently being used by your service to help smokers who wish to stop smoking?

**2 **In your experience, at what point do smokers who have quit smoking most often relapse to smoking?

**3 **What do you understand by smoking relapse prevention interventions?

### Experiences of delivering Relapse Prevention Interventions within Stop Smoking Services

**4 **Do you routinely provide relapse prevention interventions to smokers in your service?

**5 **What types? (Content)

**6 **To whom, is it to those who have already relapsed or to all smokers after the four week quit date, regardless of smoking status at the time?

**7 **For how long is the relapse prevention intervention provided for? (Duration)

**8 **What percentage of smokers who relapse to smoking take advantage of relapse prevention treatments? (Uptake)

**9 **Are your staff trained to provide relapse prevention support?

**10 **Do you collect any data so as to monitor the effectiveness of relapse prevention support you provide?

### Feasibility

**11 **What do you think about the effectiveness of......? Varenicline (champix); bupropion (zyban); extended treatment with NRT; behavioural sessions after the four week quit date; extended telephone contact after the four week quit date; (**This question depends on the answer to questions 4 and 5)**

### Challenges Experienced

**12 **Is it difficult to get smokers to attend any clinics or sessions after the four week quit date?

**13 **Are there any groups of smokers who pose a challenge in particular?

**14 **What other challenges do you face with respect to preventing relapse in smokers?

### Service Provision

**15 **What is the nature and number of sessions offered?

**16 **What is the length and timing of these sessions?

**17 **What is the actual uptake of these treatment sessions by smokers?

**18 **What kind of staff are involved in providing these services for smokers who wish to quit?

## Pre-publication history

The pre-publication history for this paper can be accessed here:


